# Functional analysis of Orco and odorant receptors in odor recognition in *Aedes albopictus*

**DOI:** 10.1186/s13071-016-1644-9

**Published:** 2016-06-27

**Authors:** Hongmei Liu, Tong Liu, Lihua Xie, Xiaoming Wang, Yuhua Deng, Chun-Hong Chen, Anthony A. James, Xiao-Guang Chen

**Affiliations:** Department of Pathogen Biology, Key Laboratory of Prevention and Control of Emerging Infectious Diseases of Guangdong Higher Education Institutes, School of Public Health, Southern Medical University, Guangzhou, Guangdong People’s Republic of China; Institute of Molecular and Genomic Medicine, National Health Research Institutes, Miaoli, Taiwan; Departments of Microbiology and Molecular Genetics, and Molecular Biology and Biochemistry, 3205 McGaugh Hall, University of California, Irvine, CA 92697-3900 USA

**Keywords:** Mosquito, Olfactory, Heterologous expression, HEK293 cells

## Abstract

**Background:**

*Aedes albopictus* is a globally invasive mosquito and a major vector of arboviruses, including dengue, Zika and Chikungunya. Olfactory-related behaviors, particularly host-seeking, offer opportunities to disrupt the disease-transmission process. A better understanding of odorant receptors (ORs) may assist in explaining host selection and location, and contribute to novel strategy of vector control.

**Methods:**

Based on previous prediction of 158 putative odorant receptors by *Ae. albopictus* genome analysis, 29 AalORs were selected for tissue-specific expression profiles in the present study. AalOrco (AalOR7), AalOR10 and AalOR88, highly expressed in female olfactory tissues, were chosen for further structure predictions as well as functional validation including calcium imaging assay in human embryonic kidney (HEK293) cells and RNA interference assay in *Ae. albopictus*. We also conducted electrophysiological and behavioral assays in mosquitoes after RNA interference of the three genes to determine their roles in host-seeking.

**Results:**

The results support previous conclusions that individual conventional (ORXs) and Orco can form heteromeric complexes to recognize odorants and respond to components of human volatiles in HEK293 cells. The reduction of AalOrco transcript levels led to a significant decrease in host-seeking and confusion in host preference. In contrast, AalOR10 and AalOR88 knockdown mosquitoes showed no significant behavioral differences compared with controls. The functions of conventional ORs at least AalOR10 and AalOR88 are abolished with inhibited expression of the Orco gene orthologs, along with the concomitant relevant olfactory behavior.

**Conclusions:**

Combining structural and functional data, we conclude that the product of the Orco gene in this mosquito is crucial for transmitting olfactory signaling and conventional ORs contribute directly to odorant recognition. Our results provide insight into the linkage between odorant receptors and host-seeking in this important vector species.

**Electronic supplementary material:**

The online version of this article (doi:10.1186/s13071-016-1644-9) contains supplementary material, which is available to authorized users.

## Background

*Aedes albopictus* (Skuse) (Diptera: Culicidae), the Asian tiger mosquito, is an important vector of arboviruses including dengue, Chikungunya [1], Zika [2] and yellow fever [3, 4]. The species has a remarkable capacity for invading new habitats worldwide, and climatic adaptation, diapause, and ability to shelter in microhabitats make it an increasingly important vector in dengue outbreaks [5, 6]. As the fact lays that *Ae. albopictus* was the sole or primary vector of recent dengue outbreaks in Europe, the Indian Ocean islands, central Africa, southern China and Hawaii [1, 6], this species is the major cause of the unprecedented outbreak occurred in China in 2014 in which more than 40,000 dengue cases were reported [4, 7]. At present, the most effective means of curbing dengue transmission is to control the vector [8]. *Aeedes albopictus* transmits dengue viruses during blood feeding [9], a behavior mediated in part by olfaction, and this offers opportunities to disrupt the transmission process.

Odorant receptors (ORs) play key roles in olfactory behaviors including a co-receptor, designated Orco (OR7), and conventional ligand-binding odorant receptors [10] (ORX). Orco is expressed in most olfactory sensory neurons (OSNs) in both adults and larvae, and is highly conserved among Diptera [11]. The conservation of its structure and expression in mosquitoes support the conclusion that Orco plays an important role in olfactory functions. However, the spatial and temporal expression profiles and functions of conventional odorant receptors, which are highly divergent and species-specific, correlate with some olfactory-mediated behavioral roles [12]. For example, AaOR4 which is significantly associated with preference for humans, is highly expressed in the antennae [13] whereas 11 conventional ORs may perceive contacting pheromones are expressed highly in non-olfactory tissues, wings and legs, in the migratory locust [14].

Multiple roles have been proposed for Orco, the first of which is that it forms a heteromeric complex with conventional ORs (ORX+ Orco) [15, 16]. For example, *An. gambiae* Orco [17], as with its *Drosophila* ortholog DOR83b [11], forms heterodimeric complexes with conventional ORs in a heterologous HEK293 expression system, and this increases the activity of the complexed conventional ORs. Orco may have a second role in which it forms a homodimer that acts as an ion channel [17–20].

ORs may have a distinct range of odor selectivity or may respond narrowly to a salient odorant [21, 22]. These findings, along with developmental and tissue-specific expression profiles, led to the hypothesis that ORs genes expressed differentially in mosquitoes were likely to be involved in host-seeking and host preference [23]. For example, AgOR1 is expressed specifically in female *An. gambiae* and has a role in host-seeking behavior [24]. AaOR4, expressed in ‘domestic’ *Aedes aegypti*, plays an important role in responding to human odors [13]_._ The identification and function of OR families in *Ae. albopictus* is limited at this time to AalOR2, which responds to indole, a volatile in human sweat [25]. Therefore, there is an urgent need to identify the full complement of OR genes in this species, especially the Orco gene, and initiate investigations into the behaviors they drive.

Twenty-nine AalORs were selected for tissue-specific expression profile, based on previous work in 158 putative odorant receptors prediction by *Ae. albopictus* genome analysis [26]. AalOR7, AalOR10 and AalOR88 were detected specifically and abundantly in female antennae, and therefore were selected for further investigation using the heterologous expression system, HEK293 cells. AalORs were expressed individually and in combination to discover their independent response as well as interactions during odor stimulation. RNA interference and behavioral assays performed on adult mosquitoes associated specific AalORs with host-seeking and preference.

## Methods

### Mosquitoes

The *Ae. albopictus* Foshan strain was obtained from the Center for Disease Control of Guangdong Province (China) [26]. This strain, which was isolated from the wild in Foshan, Guangdong Province, was maintained in an insect chamber at 27 °C with 70–80 % relative humidity and a photoperiod of 14:10 h. Larvae were fed on yeast powder and adults were maintained on a 10 % sugar solution.

### Identification and expression profiles of AalORs

In order to search highly expressed AalOR genes in the female antennae, twenty-nine AalORs were analyzed for tissue-specific expression profile, based on previous work in 158 putative odorant receptors prediction by *Ae. albopictus* genome analysis [26]. The antennae, maxillary palps, probosces, bodies of females, and heads and bodies of males were dissected from adult mosquitoes (3–5 days post-emergence). Larvae (fourth-instar) and early pupae (first day post-pupation) also were collected. Total RNA was extracted using an RNAeasy mini kit (Qiagen, Hilden, Germany), treated with the TURBO DNA-free™ Kit (Ambion, Carlsbad, CA, USA) to digest the remaining genomic DNA, and reverse-transcribed to cDNA using Prime ScriptR RTase (Takara, Otsu, Shiga, Japan). The relative expression levels of the genes were normalized to the *Ae. albopictus* β-actin gene (DQ657949). Gene-specific primers (Additional file 1: Table S1) were designed to amplify fragments > 500 base-pairs (bp) in length from the cDNA.

### RACE-PCR

cDNA was synthesized from total RNA extracted from adult mosquitoes (4–7 days post-emergence) using the SMARTer™ RACE cDNA amplification kit (Clontech, Mountain View, CA, USA). Gene-specific primers (GSPs) for 5'or 3'-end RACE are listed in Additional file 2: Table S2.

### Sequence analysis

Inferred amino acid sequences were aligned using ClustalW and a neighbor-joining tree was built using the MEGA 5.0 program [27]. Bootstrapping was calculated by the analysis of 1,000 replicates. The membrane topology of the OR sequences was predicted using HMMTOP (version 2.0) and TMHMM server (version 2.0) [10].

### Construction of expression vectors

Specific primers containing enzyme sites (*EcoR*I and *Xba*I) were designed to amplify the full-length coding sequences (CDS) of AalOrco, AalOR10, and AalOR88 from *Ae. albopictus* adults. The eGFP and DsRed coding sequences were amplified from the pIRES-eGFP and pIRES2-DsRed plasmids (Clontech, Mountain View, CA, USA), respectively, using primers containing the appropriate restriction sites. AalOR7 was cloned into the pME18s mammalian expression plasmid [16] in-frame with the eGFP coding sequence, while AalOR10 and AalOR88 were cloned into the pME18s plasmid in-frame with the DsRed coding sequence [11]. Thus, the eGFP or DsRed molecule was fused to the amino terminus of the odorant receptor protein. The resulting plasmids were sequenced to verify the primary gene structure.

### Heterologous expression of AalORs in HEK293 cells

HEK293 cells (purchased from the Chinese Academy of Sciences) were cultured in an incubator (Thermo scientific, OH, USA) at a constant temperature of 37 °C with 5 % CO_2_ and transfected transiently with AalORs using the Lipofectamine® 2000 Reagent (Invitrogen, Carlsbad, CA) [25, 28]. Expression of ORs was confirmed by RT-PCR or subcellular location after 24 or 48 h, respectively [25]. Cells were stained at 48 h after transfection (hat) with a 1:200 dilution of the DiD cell-labelling solution (Life Technologies) for 15 min at 37 °C.

### Calcium imaging assay

Cell culture medium was removed at 48 h after transfection. Cells were rinsed three times with Hank's Balanced Salt Solution (HBSS) (without Ca^2+^) and 2 μmol/l Fluo4-AM (Dojindo Laboratories, Tokyo, Japan) was added in the dark. The medium containing Fluo4-AM was removed after 30 min, and the cells rinsed three times with HBSS before the addition of fresh HBSS (containing Ca^2+^). Fluo4-AM loaded cells were cultured at 37 °C in the dark. These cells were tested by chemicals known to activate AalORs, including indole, 1-octen-3-ol, 3-methylindole and DEET (Sigma) [21, 25, 29, 30]. Indole [21, 25, 31] and 1-octen-3-ol [21] are volatiles of human sweat and activate specific ORs. 3-methylindole, a possible oviposition site volatile, activates specifically CquiOR10 [32]. DEET is the most commonly-used insect repellent and evokes electrophysiological responses of ORs [15, 29]. All odorants (≥98 % pure) were dissolved in DMSO and added to a final concentration of 10^−6^ mol/l.

Fluorescence images were acquired using a laser scanning confocal microscope (Olympus, Japan). The green fluorescence of Fluo-4 was excited at 494 nm, and the emitted fluorescence recorded at 516 nm. The Ca^2+^ level was represented as relative fluorescence changes (ΔF/Fo), where Fo is the baseline fluorescence and ΔF is the difference between the peak fluorescence caused by stimulation [33, 34]. Baseline fluorescence was taken 100 s prior to adding the odorants. The responses were quantified by the mean values of the maximal elevations (ΔF/Fo). Each odor was assayed in triplicate per dish and at least eight cells per dish were selected randomly.

### RNA interference and qRT-PCR

siRNAs of AalOrco, AalOR10, AalOR88 and GFP were synthesized by RIB BIO Co., Ltd. (China). AalOrco - siRNA sense: 5'-GCA ACA TTT GAA GGG TAT A-3'. AalOR10 - siRNA sense: 5'-GCG TTA TAT CAG CAT CAT A-3'. AalOR88 - siRNA sense: 5'-GCA ATT TGC AAG AGC AAT A-3'. Female adults (1 day post-emergence) were anesthetized with carbon dioxide and injected with siRNA (6 μg/μl, 0.5 μl) through the intersegmental thoracic membrane [35].

The transcript levels of AalOR genes were measured using qRT-PCR. Total RNA was extracted from mosquitoes at 2 days post-injection. The remaining genomic DNA was digested and cDNA synthesized as described above. Reactions were performed on a 7,500 software real-time PCR systematic using SYBR® Select Master Mix (Life, Technologies). The *Ae. albopictus* β-actin gene was used as a reference. Specific primers for qRT-PCR are listed in Additional file 3: Table S3. RNAi assays were performed in triplicate with six biological replicates. Data were calculated using the 2^−ΔΔCT^ method.

### Behavioral assays in mosquitoes

The biting assay involved female adults (1 day post-emergence) injected as described above with siRNAs targeting AalOrco, AalOR10, AalOR88 and GFP transcripts, and water. Two days after injection, 30–50 mosquitoes were fasted for 10–12 h and placed in a cage (20 × 30 × 50 cm) prior to the feeding assay. The cages were modified to have a nylon sleeve on one side [30]. We used the hands of individual human volunteers to perform this experiment, which was approved by an Institutional Review Board. The human volunteers (*n* = 2 subjects, 1 male, 1 female, aged 25–29) are authors. The hands of individual human subjects were inserted for 5 min into two cages containing siRNA-injected or control mosquitoes (siRNA-GFP and water) [13, 30, 36, 37]. The number of blood-fed mosquitoes was determined. The ratio of blood-fed mosquitoes was calculated using the following formula: blood-fed (%) = N_b_/N_t_, where N_b_ is the number of blood-fed mosquitoes and N_t_ is the total number of mosquitoes.

The host preference assay used an anesthetized mouse placed on one side of a cage, and a human hand inserted on the other side. We measured the number of mosquitoes probing the human or mouse in 5 min with the preference index = (N_h_ - N_m_)/(N_h_ + N_m_), where N_h_ is the number of mosquitoes probing humans and N_m_ the number of mosquitoes probing mice. Five to seven replications with each assay were performed at room temperature (25–28 °C) and water-injected mosquito antennae were used as controls.

### Electroantennogram recordings

Antennae of 48 h knockdown mosquitoes were excised with surgical microscissors and mounted on an electrode (Syntech Ltd., Hilversum, The Netherlands) coated with electrode gel (Spectra 360, Parker Laboratories, INC, USA) [35]. The tested odorants were dissolved in hexane to a concentration of 10 μg/μl [35] for mosquitoes. A 10 μl sample of each solution was applied to a filter paper strip and the hexane solvent evaporated before the filter paper was inserted into a Pasteur pipette. Antennae were exposed continuously to a purified air stream (5 ml/s) with a stimulus pulse for 0.5 s and an interval time of 1 min. Signals were amplified and recorded by IDAC2 (Syntech Ltd., Hilversum, The Netherlands). Antennae olfactory responses were measured as the peak amplitude caused by stimulation. Five to seven mosquitoes were tested for each odor in each group and water-injected mosquito antennae were used as controls.

### Statistical analysis

Data from qRT-PCR were analyzed by the Student’s *t*- test. Statistical analyses of differences in the other experimental results were conducted by a one-way ANOVA followed by *post-hoc* Tukey’s HSD tests (homogeneity of variance: *P* > 0.05) or Dunnett T3 tests (homogeneity of variance: *P* < 0.05).

## Results

### Identification and expression profiles of AalORs

In order to search for highly expressed AalOR genes in the female antennae, twenty-nine AalORs were selected for tissue-specific expression profile. Of these, olfactory tissues include antennae, maxillary palps and proboscises, and heads, bodies are non-olfactory tissues, and larvae and pupae represent different growing stages. RT-PCR showed that AalOR genes were expressed highly divergent in different tissues and stages. AalORs 7, 10, 14, 45, 59, 88 and 105 were highly expressed in female olfactory tissues but not in male heads (Additional file 4: Figure S1).

AalOrco (AalOR7), the *Ae. albopictus* olfactory receptor co-receptor (Orco) ortholog, is expressed highly in female olfactory tissues including the antennae. AalOR10 and AalOR88 also are expressed highly in female antennae. The *Culex quinquefasciatus* ortholog of AalOR10, CquiOR10, is involved in the selection of oviposition sites and its transcripts are enriched in antennae [38]. AaOR88 transcripts are > 23-fold more abundant is non-blood-fed female versus male antenna, and may be involved in sex-specific behaviors, such as blood-feeding [39]. The observed *Ae. albopictus* expression profiles and previous reports on orthologs in other mosquitoes prompted us to analyze further AalOrco, AalORs10 and 88. The full-length of AalOrco, AalORs10 and 88 were amplified using RACE-PCR, and proteins were showed in the Additional file 5.

### Phylogenetic analysis and membrane topology

**Fig. 1:**
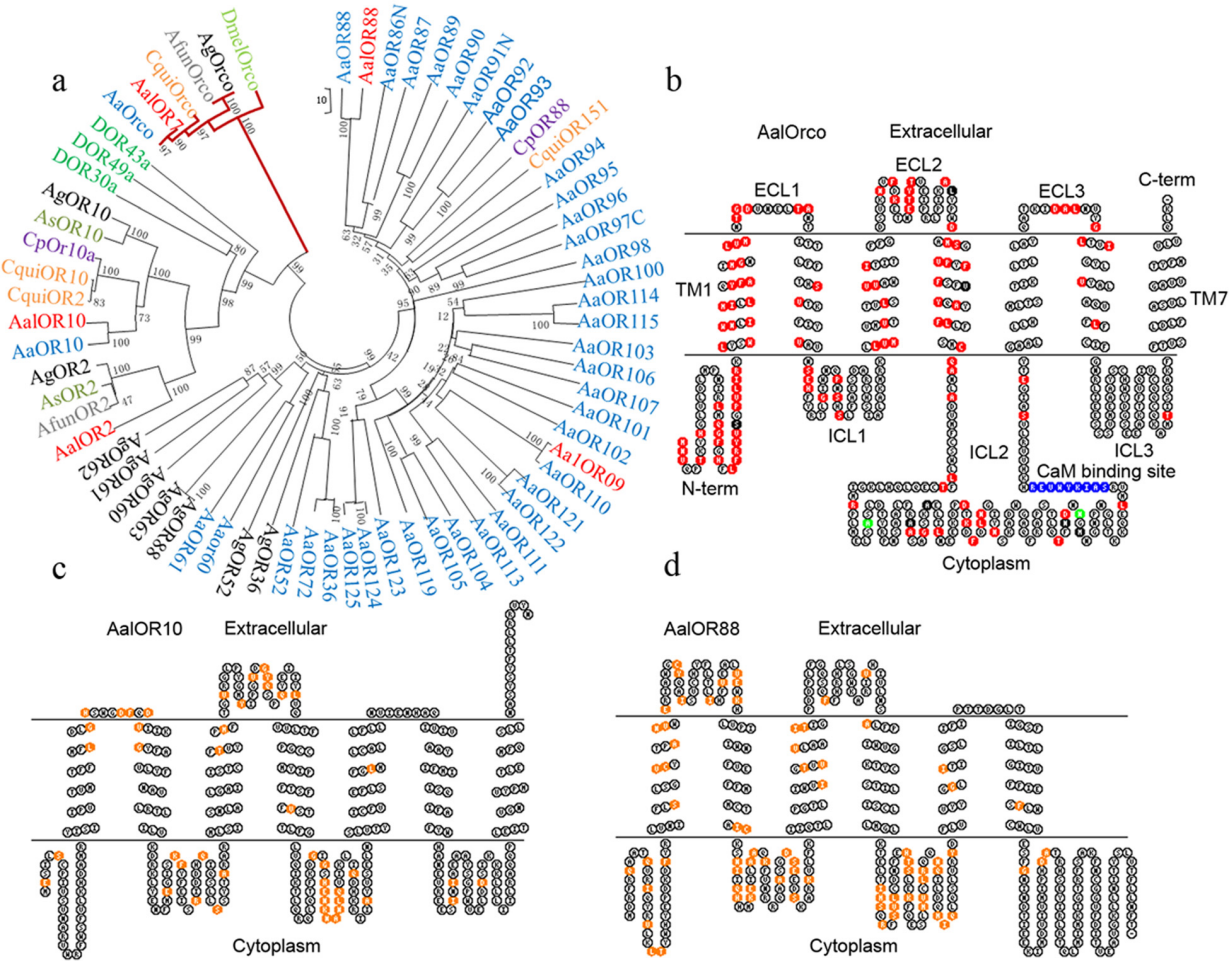
Phylogenetic relationships and transmembrane regions of four representative AalOR genes. **a** A neighbor-joining tree with AalOR 2, 7, 10 and 88 based on amino acid sequence alignment and constructed with MEGA5 using ClustalW. *Key*: *Aedes albopictus*, AalORs (*red*); *Anopheles gambiae*, AgORs (*black*); *Culex quinquefasciatus*, CquiORs (*orange*); *Ae. aegypti*, AaORs (*blue*); *Cx pipiens pipiens*, CpORs (*violet*); *Drosophila melanogaster*, DOR83b (*green*); *An. funestus*, AfunORs (*gray*); and *An. stephensi* AsORs (*olive*). **b**-**d** Transmembrane regions of AalORs predicted using HMMTOP and TMHMM. **b** The *blue* circles represent the CaM binding site, *red* circles indicate amino acids differing from the Dmel Orco ortholog, *green* circles indicate amino acids differing from AeOR7, and *black* circles represent those amino acids differing in both DmelOrco and AeOR7. **c**, **d** *Orange* circles represent amino acids that differ from the respective *Ae. aegypti* ortholog

A phylogenetic tree constructed with amino acid sequences for *Ae. aegypti*, *An. gambiae*, *An. funestus*, *Culex pipiens pipiens*, *Drosophila melanogaster*, *Culex pipiens pipiens* and *Cx. quinquefasciatus* shows as expected that the co-receptor subfamily (AalOrco, AaOrco, AgOrco, CquiOrco and DmelOrco) is clustered in one branch with clear orthologous relationships among the species (Fig. 1a). AalOrco is most similar (~99 % identity) to the *Ae. aegypti* ortholog, AaOR7. AalOR10 and AalOR88 were identified as conventional odorant receptors and most similar to AaOR10 (96 % identity) and AaOR88 (81 % identity), respectively.

Insect OR proteins have six or seven transmembrane (TM) domains and an intracellular amino-terminus [40]. Membrane topology predictions of AalOrco and AalOR10 show that they belong to the TM7 class while AalOR88 is a TM6 protein (Fig. 1b–d). Analysis of the primary amino acid sequence of AalOrco shows that it shares the highly-conserved intracellular loop 3 (ICL3), TM6 and TM7 regions with other Orco proteins and a putative calmodulin (CaM) binding site (^329^SAIKYWVER^337^) identified in DmelOrco (^336^SAIKYWVER^344^), in the ICL2 domain (Fig 1b). This sequence conservation supports our hypothesis that the AalOrco TM6 and ICL3 regions could form a channel gate as is seen with DmelOrco [41, 42], and that the ICL3 and TM7 regions could interact with the TM7and ICL3 regions of conventional ORs to form complexes and participate in odor signal transduction [42]. This conservation of structure also may account for the ability of Orcos from different insects to substitute functionally for one another. AalOR10 and AalOR88 do not have the putative CaM binding site and channel gate sequences. Recent studies on structural features and function of Orco show that ICL3 is important for Orco channel activation [43], and CaM (in ICL2) activity affects the function of Orco channels [44]. Extracellular loop 2 (ECL-2) and TM4 are essential for the odorant response-specificity of AgOR15 [45]. It is generally believed that the ICL3 regions of Orco and ORXs interact [10], which maybe affect the function of Orco and ORXs complex [42].

### Heterologous expression of AalOrco, AalOR10 and AalOR88 in human embryonic kidney 293 cells

**Fig. 2:**
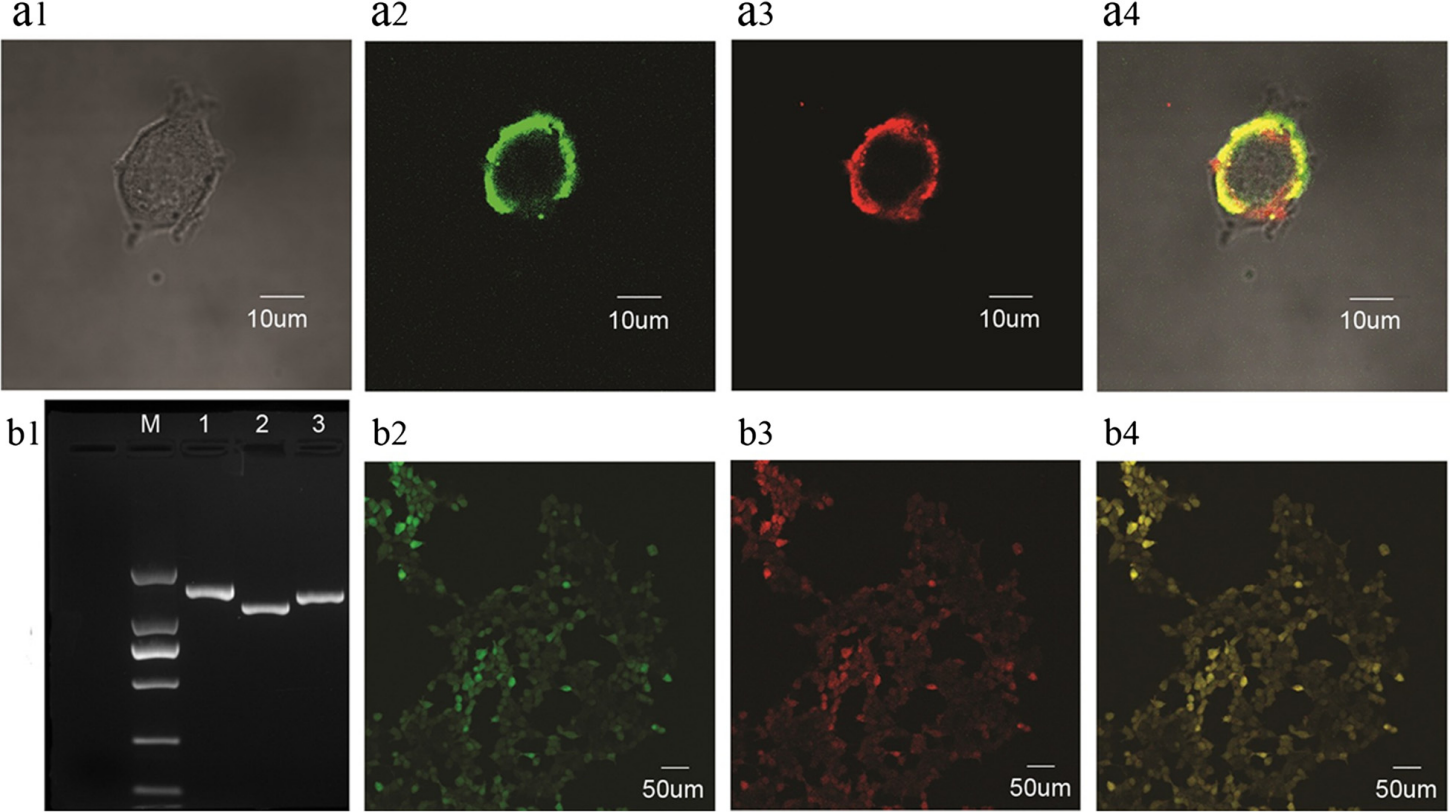
Subcellular localization of expressed mosquito odorant receptors in HEK293 cells. **a1** HEK293 cells (no fluorescence). **a2** Same as a1 with AalOR7 expression of AalOR7-eGFP. **a3** Same as a1 with AalOR7-eGFP-transfected cells stained with DID cell-labelling solution. **a4** Same as A1 with AalOR7 expressed in HEK293 cells stained with DID membrane stain. **b1** AalORs transcripts detected by RT-PCR in HEK293 cells 24 h after transfection. Lane M: molecular weight marker in the 2,000 bp series; Lane 1: AalOR7; Lane 2: AalOR10; Lane 3: AalOR88. **b2** HEK293 cells expressing AalOR7-eGFP. **b3** HEK293 cells expressing AalOR10-DsRed. **b4** HEK293 cells co-expressing AalOR7-eGFP and AalOR10-DsRed

AalORs transcripts are detected in HEK293 cells at 24 hat (Fig. 2 b1). Furthermore, the corresponding proteins are localized to the plasma membrane by 48 hat in cells transfected individually (Fig. 2 a2-a4) and may be co-localized in combination (AalOrco-eGFP and AalOR10-DsRed; Fig 2 b2-b4), although it is difficult to quantify the proportion of OR protein abundance in the latter.

AalOrco could form a channel gate in the plasma membrane, and the ORX + Orco heteromeric complex (AalOrco/AalOR10) might act as an odorant-gated cation channel with ionic permeability mostly for Ca^2+^. Chemicals known to activate *Ae. aegypti* ORs include indole [21], 1-octen-3-ol [21], 3-methyindole [35] and N,N-diethyl-3-methylbenzamide (DEET) [15, 29]. Transfected HEK293 cells may respond by increasing intracellular calcium [28, 46] when exposed to these chemicals.

**Fig. 3:**
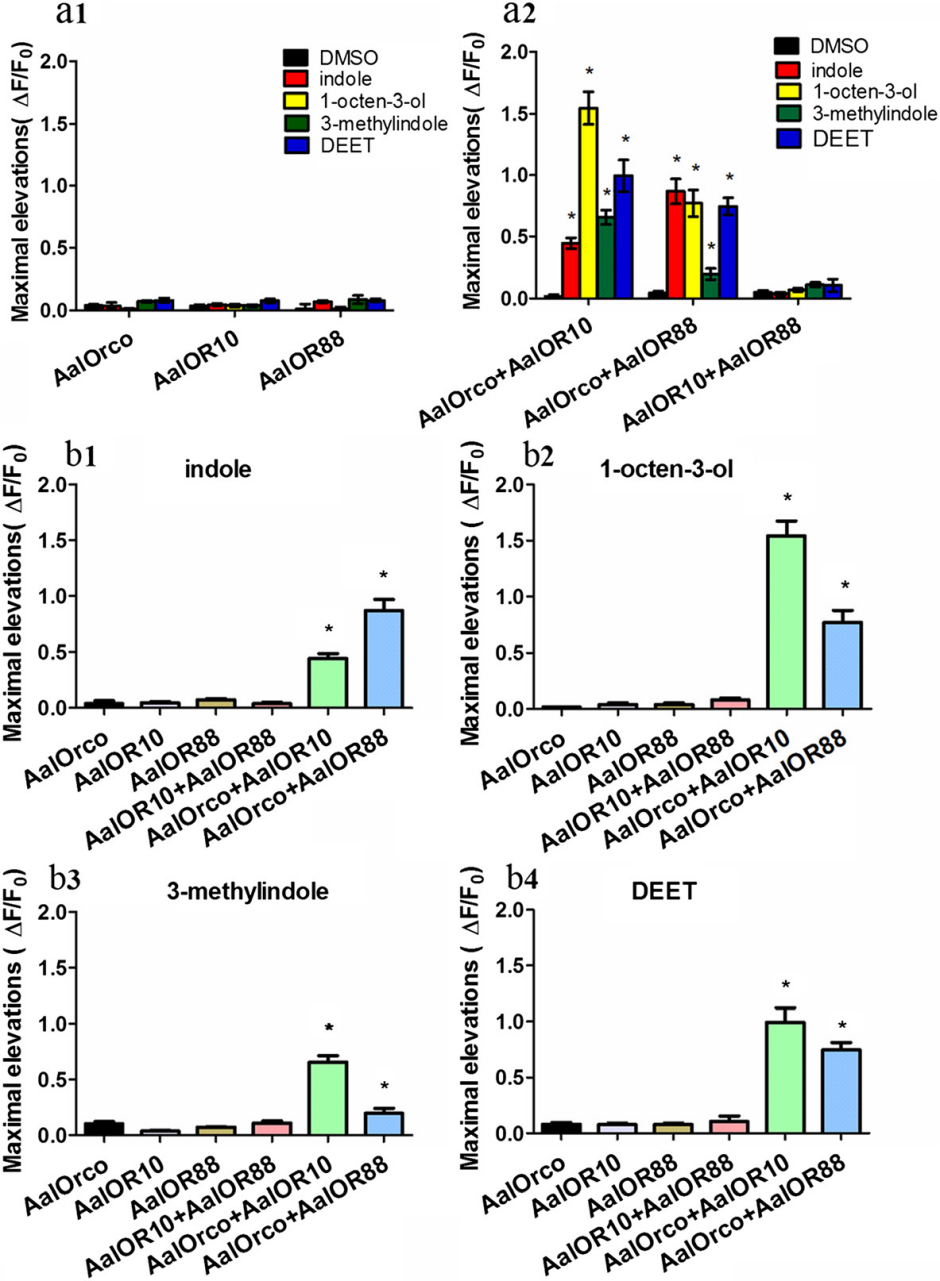
Odorants activate calcium entry in HEK293 cells expressing AalORs. Ca^2^+ levels are represented as ΔF/Fo, where Fo is the baseline fluorescence signal intensity before stimulation, and ΔF is the difference in peak fluorescence caused by stimulation. The responses were quantified by the mean values of the maximal elevations (ΔF/Fo). The maximal intracellular calcium concentrations activated by indole (*red*), 1-octen-3-ol (*yellow*), 3-methyindole (*green*), DEET (*blue*), and DMSO (control, *black*) are provided. Maximal intracellular calcium concentrations occurred in HEK293 cells expressing AalOR7 (*F*_(4,189)_ = 15.136, *P* < 0.0001, Dunnett T3 *vs* DMSO, indole: *P* = 1.000, 1-octen-3-ol: *P* = 0.128, 3-methyindole: *P* = 0.103, DEET: *P* = 0.310), AalOR10 (*F*_(4,199)_ = 1.654, *P* = 0.162), AalOR88 (*F*_(4,200)_ = 4.679, *P* = 0.002, Dunnett T3 *vs* DMSO, indole: *P* = 0.569, 1-octen-3-ol: *P* = 1.000, 3-methyindole: *P* = 0.549, DEET: *P* = 0.442) (**a1**) AalOR7+ AalOR10 (*F*_(4,121)_ = 76.193, *P* < 0.0001), AalOR7+ AalOR88 (*F*_(4,128)_ = 47.871, *P* < 0.0001), and AalOR10+ AalOR88 (*F*_(4,151)_ = 1.733, *P* = 0.146) (**a2**) upon stimulation with indole, 1-octen-3-ol, 3-methyindole and DEET. The differences in single AalORs and complexes responding to the same odorant were analyzed: **b1** Indole (*F*_(5,197)_ = 27.481, *P* < 0.0001); **b2** 1-octen-3-ol (*F*_(5,186)_ = 38.934, *P* < 0.0001); **b3** 3-methyindole (*F*_(5,240)_ = 24.641, *P* < 0.0001); and **b4** DEET (*F*_(5,175)_ = 26.955, *P* < 0.0001). These results are representative of three independent experiments (one-way ANOVA test, Dunnett T3). Bars represent the means ± SD. *P < 0.05

Calcium imaging experiments [11, 21, 25, 28] showed no significant differences (measured as relative fluorescence changes, ΔF/Fo) compared to DMSO controls in intracellular calcium concentration in HEK293 cells expressing only AalOrco, AalOR10 or AalOR88 stimulated with the test chemicals (Fig. 3 a1). We interpret these results to indicate that individual AalOR proteins respond weakly, if at all to odorants. However, cells co-expressing AalOrco and AalOR10 responded strongly to indole, 1-octen-3-ol, 3-methyindole and DEET (Fig. 3 a2). This response profile in HEK 293 cells suggests that AalOR10 is more sensitive to 1-octen-3-ol than indole. However, several mosquito OR10 orthologs are clearly tuned to indole, with very little response to 1-octen-3-ol [31]. A similar contrast exists for AaOR4, changes in the AaOR4 coding region affect response to sulcatone [13]. A D466E DmelOrco substitution mutant was two-fold more sensitive to the agonist VUAA1[19]. The differences in coding region amongAalOR10 with other OR10 orthologs maybe affect protein function. Cells co-expressing AalOrco and AalOR88 also are activated by the tested odors (Fig. 3 a2). In contrast, cells co-expressing AalOR10 and AalOR88 did not respond to any odorant (Fig. 3 a2). Thus, the conventional AalORs appeared to detect odorant stimulations only in the presence of AalOrco.

The previous findings and conclusions are supported by experiments in which cells expressing single AalORs or complexes respond to the same odorant. Responses of cells co-expressing AalOR10 and AalOR88 were weak, similar to those of cells expressing AalOR10 or AalOR88 alone (Fig. 3 b1-b4). However, co-expression of AalOR10 or AalOR88 with AalOrco produced responses that are significantly different when compared to the single AalORs. The results support the conclusion that AalOrco acts in synergy, possibly by forming a complex, to comprise a functional olfactory receptor and respond to odorants and transmit odor signals.

### RNA interference and mosquito behavioral experiments

**Fig. 4:**
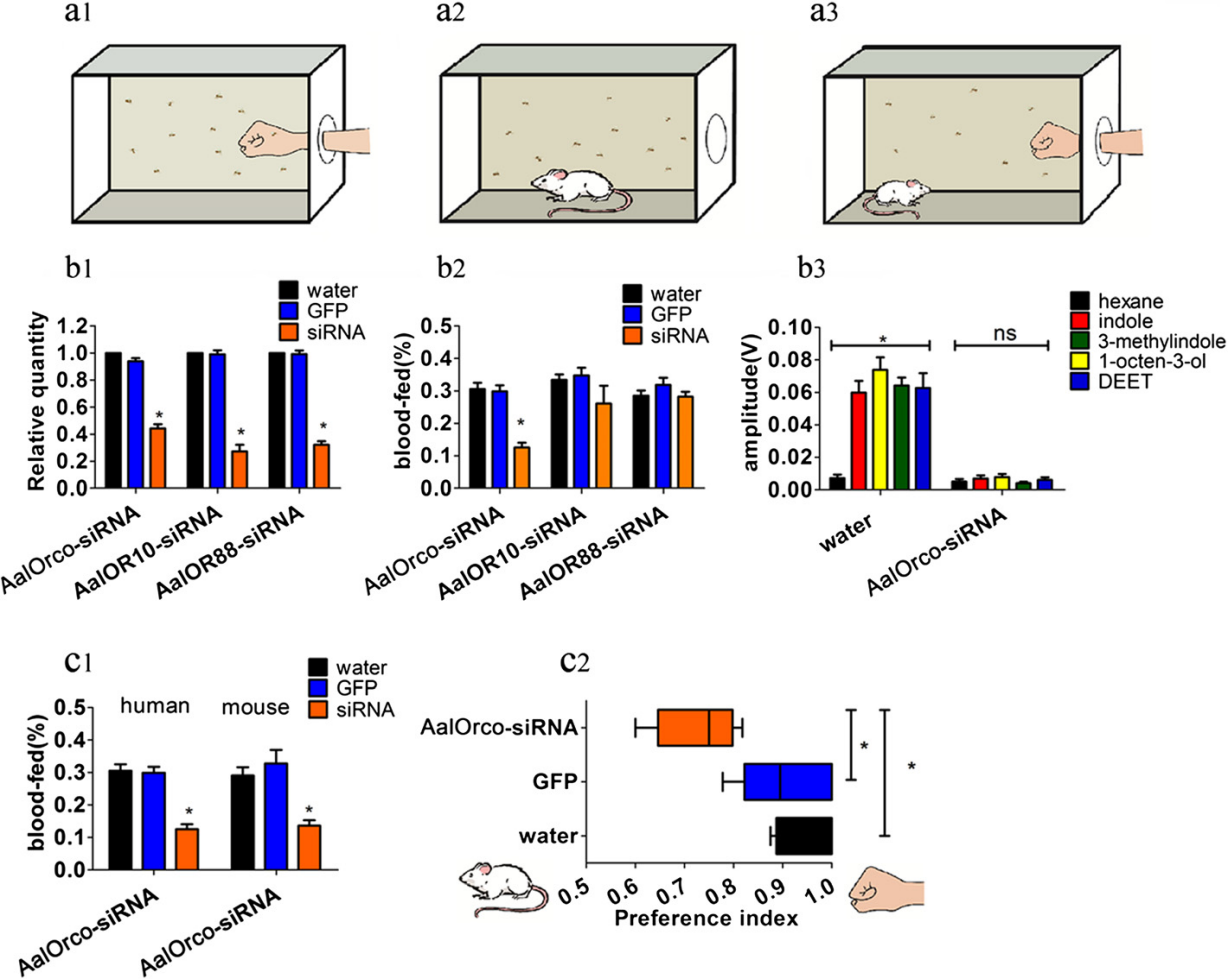
Behavioral assays of RNAi-ablated mosquitoes. **a1**, **a2** Biting assay scheme. **a3** Host preference assay scheme. **b1** Transcript abundance of AalORs was reduced significantly after 48 h siRNA injections (AalOR7-siRNA-treated mosquitoes: *t*_(10)_ = 13.191, *P* < 0.0001; AalOR10-siRNA-treated mosquitoes: *t*_(10)_ = 12.490, *P* < 0.0001; AalOR88-siRNA-treated mosquitoes: *t*_(10)_ = 18.275, *P* < 0.0001). **b2** AalOR7-siRNA-injected mosquitoes showed a significantly lower blood-feeding rate compared to the control (*F*_(2,15)_ = 32.183, *P* < 0.0001). AalOR10-siRNA-injected mosquitoes (*F*_(2,15)_ = 1.690, *P* = 0.218) and AalOR88-siRNA-injected mosquitoes (*F*_(2,15)_ = 1.361, *P* = 0.286) showed no significant differences. **b3** Electroantennograms of mosquito antennae stimulated with odorants. Water-injected mosquito antennae responded strongly to odorants (*F*_(4,20)_ = 15.766, *P* < 0.0001) while AalOR7-siRNA-treated mosquitoes (*F*_(4,20)_ = 0.808, *P* = 0.532) did not respond to any odorant. **c1** Preference index for human or mouse (*F*_(2,12)_ = 16.724, *P* = 0.002). **c2** Host preference experiment. AalOR7-siRNA-treated mosquitoes showed a statistically significant lower preference for humans. (*F*_(2,12)_ = 9.738, *P* = 0.003) (one-way ANOVA test, Tukey’s HSD tests or Dunnett T3). Bars represent the means ± SD (*n* = 5–7). *P < 0.05

AalORs transcript abundances are reduced ≥ 50 % following injections of gene-specific siRNAs when compared to mosquitoes injected with water and GFP-siRNA (Fig. 4b1). A modified assay using the hands of volunteers (Fig. 4 a1) measured the number of blood-fed mosquitoes in 5 min and this was used to calculate the blood-feeding rate. AalOrco-siRNA injected mosquitoes have a significantly lower blood-feeding rate compared to the controls (Fig. 4 b2). We interpret this to indicate that AalOrco-siRNA-treated mosquitoes have difficulties in detecting a host. AalOR10-siRNA- and AalOR88-siRNA-injected mosquitoes show no significant differences compared to the controls (Fig. 4 b2). We interpret this to indicate that the functions of these conventional ORs could be complemented by other factors, including other ORs, or that they may not be involved in host-seeking. AalOrco siRNA-injected mosquito antennae exhibit a lack of sensitivity to all tested odors when compared to water-injected controls (Fig. 4 b3, and Additional file 6: Figure S2), further supporting a role AalOrco in host-seeking.

*Aedes albopictus* prefers human hosts, but also can feed on a large variety of animals, including mice [3, 47, 48]. Host preference experiments using humans and mice show that AalOrco -siRNA injected mosquitoes were diminished significantly in their ability to detect either (Fig. 4a1, a2, c1). Host preference experiments (Fig. 4 a3) indicate that both GFP-siRNA and water-injected mosquitoes strongly prefer human, whereas those treated with AalOrco -siRNA have a statistically-significant lower bias for humans (Fig. 4c2).

## Discussion

Here we reported the high expression of AalOrco, the *Ae. albopictus* Orco ortholog, AalOR10 and AalOR88 in adult female antennae and their involvement in olfactory functions. AalOrco was predicted to form an ion channel based on its primary amino acid structure, which is consistent with what was reported previously in *Drosophila* and other mosquito species [42, 49]. Conventional ORs, AalOR10 and AalOR88 were predicted structurally to detect odorants. The structural distinctions between Orco and the conventional ORs support the logic that they might affect olfactory functions in different ways. Indeed, we showed that AalOR10 and AalOR88 respond to odor stimulation in HEK293 cells in the presence of AalOrco alone. However, neither Orco nor the conventional ORs responded to odorants when expressed independently in the heterologous system. These results provide further evidence supporting the hypothesis that ORX and Orco form a heteromeric complex to recognize odorants and respond to stimulation. Moreover, AalOR10 and AalOR88 responded with different sensitivity to each odor. The functional divergence between conventional ORs might be correlated with different behaviors.

Both AalOR10 and AalOR88 were found to be more sensitive to human volatiles, indole and 1-octen-3-ol in the heterologous expression system. Female mosquitoes rely on environmental attractants to seek a host. High concentration of Indole presents in human volatiles [50, 51] despite its wide existence in nature, and 1-octen-3-ol can attract mosquitoes from far distance in the field [52]. Both molecules were proved to contribute to host detection. Contrary to this expectation, our research showed that mosquitoes were able to seek out a host when AalOR10 or AalOR88 were ablated by transcript-specific siRNAs. Such result supports the conclusion that the function of these ORs could be complemented, most likely by other ORs, but they also might not be involved in host-seeking. Recent studies support the proposal that host-seeking is mediated by multiple ORs, including AaOR4, which is linked tightly to human odor-seeking [13], and AgOR2 [21] and AalOR2 [25], both respond preferentially to indole. Host-seeking behavior may not rely on individual conventional ORs but result from the cumulative effects of multiple ORs. However, the reduction of AalOrco transcript levels produced obvious defects not only in detecting but also in discriminating a host. Thus, the results support a critical role for this gene and its product in olfactory activity. Female mosquitoes with mutations in the *Ae. aegypti* Orco ortholog lose their preference for human odors [30], and analogous mutations in *Drosophila* lose responses to many odorants [53]. Inhibiting Orco expression then abolishes the functions of conventional ORs at least AalOR10 and AalOR88 along with the relevant behavior. The data also support the hypothesis that conventional ORs contribute directly to the sensitivity of different odors, while Orco plays a crucial role in forming a ligand-gated ion channel to generate signals to induce behavioral responses, including host-seeking and host selection.

## Conclusions

In summary, we identified AalOrco, AalOR10 and AalOR88 are the functional odorant receptors in *Ae. albopictus* and results provide further support that co-expression of conventional ORs with Orco is required for normal response to odors. Moreover, Orco is the crucial gene for olfactory signaling. This gene is a key to olfactory behavior and may prove a useful target for blocking host-seeking.

## Abbreviations

CaM, calmodulin; CDS, the full-length coding sequence; ECL, extracellular loops; GSP, gene-specific primer; HBSS, Hank's Balanced Salt Solution; HEK293 cell, human embryonic kidney 293 cell; ICL, intracellular loop; OR, odorant receptor; OSN, olfactory sensory neuron; TM, transmembrane

## Additional files

Additional file 1: Table S1. List of oligonucleotide primers for RT-PCR. (XLS 21 kb) Additional file 2: Table S2. List of oligonucleotide primers for RACE. (XLS 18 kb) Additional file 3: Table S3. List of oligonucleotide primers for qRT-PCR. (XLS 18 kb) Additional file 4: Figure S1. RT-PCR analyses of 29 of AalOR transcripts in different tissues. *Abbreviations*: Actin, β-actin; L, larvae; P, pupae; An, female antennae; Mp, female maxillary palp; Pro, female proboscis; FB, female body; MH, male head; MB, male body (TIF 1014 kb) Additional file 5: Proteins of AalOrco, AalOR10 and AalO88. (PDF 6 kb) Additional file 6: Figure S2. Electroantennograms recordings of siRNA-treated mosquito antennae stimulated with odorants. AalOR7-siRNA-injected mosquito antennae revealed a lack of sensitivity to all tested odors at 48 h post injection. Water-injected mosquito antennae showed strong odor responses. (TIF 156 kb)
