# Supplementary figures and images for: Functional analysis of Orco and odorant receptors in odor recognition in Aedes albopictus
Source: Parasit Vectors. 2016 Jun 27;9:363. doi: 10.1186/s13071-016-1644-9 (PMC4924234; doi:10.1186/s13071-016-1644-9)

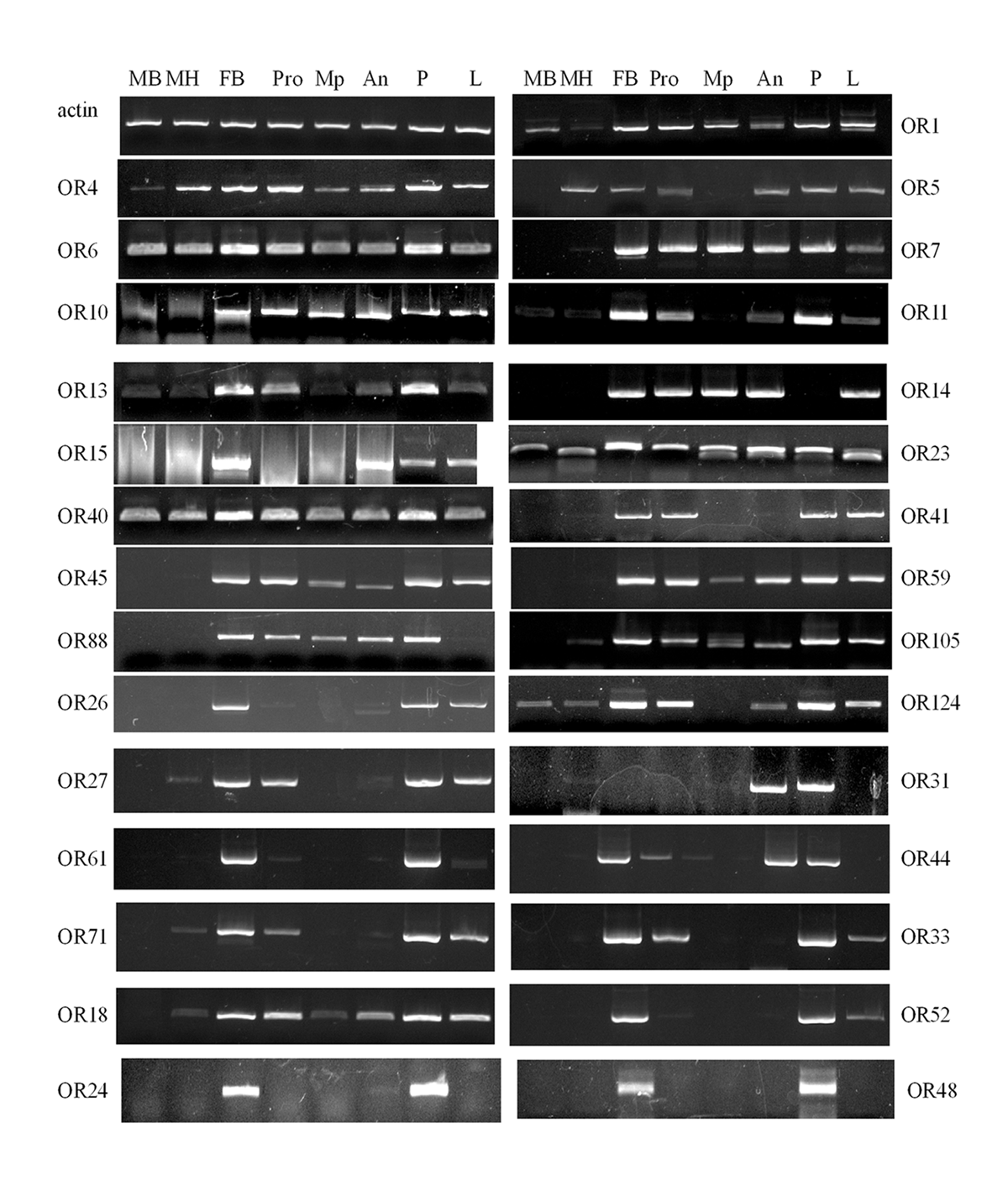

Supplement: Additional file 4: Figure S1. — RT-PCR analyses of 29 of AalOR transcripts in different tissues. Abbreviations: Actin, β-actin; L, larvae; P, pupae; An, female antennae; Mp, female maxillary palp; Pro, female proboscis; FB, female body; MH, male head; MB, male body (TIF 1014 kb) [file 13071_2016_1644_MOESM4_ESM.tif]

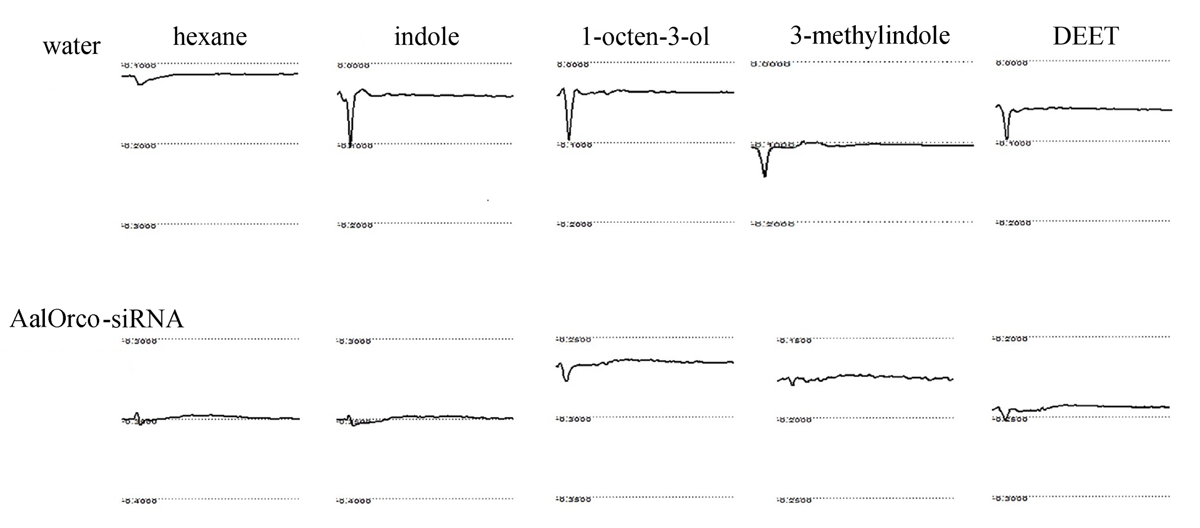

Supplement: Additional file 6: Figure S2. — Electroantennograms recordings of siRNA-treated mosquito antennae stimulated with odorants. AalOR7-siRNA-injected mosquito antennae revealed a lack of sensitivity to all tested odors at 48 h post injection. Water-injected mosquito antennae showed strong odor responses. (TIF 156 kb) [file 13071_2016_1644_MOESM6_ESM.tif]
