# Supplementary material for: Functional analysis of Orco and odorant receptors in odor recognition in Aedes albopictus
Source: Parasit Vectors. 2016 Jun 27;9:363. doi: 10.1186/s13071-016-1644-9 (PMC4924234; doi:10.1186/s13071-016-1644-9)
Supplement: Additional file 5: — Proteins of AalOrco, AalOR10 and AalO88. (PDF 6 kb) [file 13071_2016_1644_MOESM5_ESM.pdf]

Protein of AalOrco

MNVQPTKYHGLVLDLMPNIRLMQGFGHFLFRYVSGPVLIRKLYSWWNLIMIL
LQYFAIMGNLVMNTGDVNELTANTITTLFFTHSVTKFIYVAVNSEHFYRTLGI
WNQPNSHSLFAESDARYHSIALAKMRKLLVMVMVTTVLSVVAWITITFFGDS
VKNVFDKETNETYTVEIPRLPIKALYPWDAMSGVPYFFSFVYQAYFLLFSMC
QANLADVMFCSWLLFTCEQLQHLKGIMRPLMELSASLDTYRPNSAALFRAAS
AGSKAELILNEEKDPDTKDFDLNGIYNSKADWGAQFRAPSTLQTFNDNNGM
NGNPNGLTKKQELMVRSAIKYWVERHKHVVRLVSAIGETYGAALLLHMLTS
TIKLTLLAYQATKIDALNVYGLTVIGYLVYALAQVFLFCIFGNRLIEESSSVME
AAYSCHWYDGSEEAKTFVQIVCQQCQKAMTISGAKFFTVSLDLFASVLGAV
VTYFMVLVQLK-

Protein of AalOR10

MESILSCPIVSVNARVWRFWSFVLKHDAMRYISIIPVTVMTFFMFLDLGHSWG
DFQDVIIKGYFAVLYFNAVLRTLILVKDRKLYENFMEGISKFYFEISRIDDHQI
QSLLSSYTARARMLSISNLALGAIISTCFTVYPMFTGVRGLPYGMYIPGVDGY
QSPQYEIIYLVQVVLTFPGCCMYIPFTSFFVSTTLFGLVQIKTLQRQLQTFKDGI
GSHENKNANLQVIKLIQDHKRIIAYVSELNSLVTYICFVEFLSFGLMLCALLFL
LNVIENHAQIVIVAAYIFMIISQIFAFYWHANEVREESINIAEAAYSGPWVELD
DSIKKKLLLIILRAQQPLEITVGNVYPMTLEMFQSLLNASYSYFTLLRRVYN

Protein of AalOR88

MDRFRQAATQVRIQYRVLKLTYWHYEPDKDFYRLVNIFLSLSGVCYTPANVW
ERIAWGCYQAISVCHYFLTLIHFVEALVEWKHLVPIIWNFVEFFMMCTAICKSN
IIQHHMKEILAAQKFVNRRQAASGDSEEDSKARKVLFKIIQTLPGTLIWVIGTV
VVLAAITIGPDDPLFGQSQFFHRHLSGWKARIVHIVLNSALFPIWVGKTYCSTI
LISCLLMGLKTELTIMSQRFSVLCDNFEHTSLEKFQESLMVCLRQQNQILDQMI
QLQSLVRYDFLVVYYGGLITIGSLIFIPTTDGLTIGSFILTLGITVFFIESFLWCWL
VESFGNINDTIEEKIMDVIVMIPHDATRHSEYIRLRTQLMIMKIGTRFSAKFNCG
GVFDITVEAVGKLLNITYSLVTFLLNFT-
